# Potential of nicotinamide adenine dinucleotide (NAD) for management of root-knot nematode in tomato

**DOI:** 10.21307/jofnem-2021-094

**Published:** 2021-11-11

**Authors:** Homan Regmi, Noor Abdelsamad, Peter DiGennaro, Johan Desaeger

**Affiliations:** 1 Entomology and Nematology Department, University of Florida, Gulf Coast Research and Education Center (GCREC), Wimauma, FL, 33598; 2 United States Department of Agriculture-Agriculture Research Services (USDA-ARS), San Joaquin Valley Agricultural Sciences Center, Parlier, CA, 93648; Entomology and Nematology Department, University of Florida, Gainesville, FL, 32608

**Keywords:** Induced systemic resistance, Management, *Meloidogyne hapla*, *M. incognita*, Plant disease, Systemic acquired resistance, Tomato

## Abstract

Nicotinamide adenine dinucleotide (NAD) has been shown to induce plant defense responses to different plant pathogens, including reducing northern root-knot nematode, *Meloidogyne hapla*, penetration and increasing plant mass in tomato. We wanted to further evaluate NAD that are effective against the more economically important species, *M. incognita* and whether NAD treatments of tomato seedlings in transplant trays can protect plants in the field. Different NAD concentrations (1  mM, 0.1  mM and 0.01  mM) and three application timings (pre; post; pre and post inoculation) were evaluated in growth room and greenhouse trials. The highest tested NAD concentration (1  mM) suppressed second-stage juveniles (J2) infection for all three application methods. Root gall ratings at 30 days after inoculation (DAI) were also suppressed by 1  mM NAD compared to the other two concentrations, and egg mass number was significantly suppressed for all concentrations and application timings compared to the non-treated control. The rate of 1  mM NAD for all three application timings also improved plant growth at 30 DAI. Long-term effects of 1  mM NAD (pre, pre + post, or post applications) on nematode infection, growth and yield of tomato were evaluated in two additional experiments. All NAD applications suppressed root galls after 60 days, but only the pre + post 1  mM NAD application suppressed gall severity at 105 days, as well as suppressed egg counts by 50% at 60 DAT. No significant difference in plant biomass and fruit yield after 105 days was observed among the treatments. Two field trials were conducted in spring and fall 2020 using tomato seedlings (cv. HM 1823) treated with two different NAD concentrations (1  mM and 5  mM in spring; 5  mM and 10  mM in fall) and transplanting seedlings in fumigated (chloropicrin  ±  1,3-dichloropropene) and non-fumigated plastic-mulch beds. No significant impact of NAD in terms of reducing RKN severity or overall tomato growth and production was seen in fumigated beds, but in non-fumigated beds 5  mM NAD slightly increased early fruit yield in spring, and 10  mM NAD reduced root-knot soil populations in fall.

Non-chemical approaches to combat various pests and pathogens in agriculture including plant-parasitic nematodes are desirable as they are safer to humans and the environment. However, use of chemical fumigants, especially combinations of 1,3-D and chloropicrin are the standard practice in Florida tomato (*Solanum Lycopersicon* L.) fields to combat the problem of both RKN and soilborne pathogens (Santos et al., 2006; Minuto et al., 2006; Desaeger et al. 2017). When available, host resistance is safe, cheap and the overall preferred method to manage pests and diseases. Root-knot nematodes (RKN), *Meloidogyne* spp., are among the most damaging pests causing significant economic loss in a range of agricultural crops including tomato. Tomato is considered one of the best hosts of RKN and results in yield losses ranging from 25 to 100% (Seid et al., 2015). Fortunately, tomato is one of the few vegetable commodities where resistance against RKN is commercially available. The resistance is governed by a single dominant gene, *Mi*, which is effective against the most common tropical and sub-tropical species of RKN (*M. incognita*, *M. javanica* and *M. arenaria*. However, it is not effective against other RKN species that are common in Florida such as *M. hapla*, *M. enterolobii*, *M. floridensis* or *M. haplanaria* (Crow and Duncan, 2018). Also, in Florida, where mostly fresh market varieties are grown, the most popular varieties do not tend to have the *Mi* gene (Regmi and Desaeger, 2019). In addition, host resistance, especially a monogenic one such as *Mi*, has a high tendency to be broken down by the virulent pests over time (Thakur, 2007) which has been observed in most areas where *Mi* cultivars are commonly grown (Kaloshian et al., 1996; Tzortzakakis et al., 2014).

More recently, research has shown that plant metabolites, like salicylic acid (SA), jasmonic acid and ß-aminobutyric acid, can prime plant defense pathways and stimulate innate immunity of a plant against a broad range of pests and pathogens (Kessmann et al., 1994; Smith et al., 2009; Barilli et al., 2015). Among several plant immunity activators, acibenzolar-S-methyl, a derivative of benzothiadiazole, was the first commercially available chemical elicitor which has been successfully integrated in crop management programs (Reddy, 2012). ASM is a chemical activator which activates systemic acquired resistance against plant diseases by mimicking SA in a signal transduction pathway (Matheron and Porchas, 2002). Actigard^TM^ (Syngenta, Greensboro, NC, United States) is based on this biochemical, and is used in tomato cultivation targeting especially bacterial speck caused by *Pseudomonas syringae* and bacterial spot disease caused by *Xanthomonas vesicatoria* (Graves and Alexander, 2002). Plants synthesize several different secondary metabolites, usually specific to a family or species of plant, that can act as natural pesticides or signals against a wide range of pathogens (Pétriacq et al., 2013). In addition to secondary metabolites, primary metabolism and its key factors are also considered to be important, since they play important roles in mitochondrial Reactive Oxygen Species (ROS) production and in maintaining the production of secondary metabolites (Gleason et al., 2011). The primary metabolites are present in every living cell and have major roles in the life of the organism while secondary metabolites are derived from primary metabolites and do not have paramount significance to continue life in living organism (Thirumurugan et al., 2018). Nicotinamide adenine dinucleotide (NAD), a primary metabolite, is one such chemical elicitor that regulates plant defense responses to different biotic stresses (Louw and Dubery, 2000; Pétriacq et al., 2012; Zhang and Mou, 2009).

Previous investigations on the use of NAD against *M. hapla* showed that a drench application of 5 mM 24 hr before nematode inoculation, significantly induced defense response pathways, suppressed infective juvenile penetration and increased plant mass in tomato (Abdelsamad et al., 2019). We wanted to further evaluate the potential of NAD to treat and prime tomato seedlings in transplant trays, prior to planting in the field. Also, in the previous work, *M. hapla* was used which is not economically important in Florida as the thermophilic species like *M. javanica*, *M. incognita* and *M. arenaria*. Therefore, we wanted (i) to evaluate the potential of NAD against a thermophilic RKN species, (ii) evaluate the optimal concentration and application timing of NAD, and (iii) determine the length of protection and (iv) evaluate transplant tray treatments for protection of tomato under natural field conditions.

## Materials and methods

### General procedures for growth room and greenhouse experiments

Growth room experiments were conducted at the University of Florida’s Entomology and Nematology Department, Gainesville, Florida during September–December 2018 and greenhouse and field experiments were carried out at University of Florida’s Gulf Coast Research and Education Center, Wimauma, Florida between February 2019 and December 2020. A popular tomato cultivar in Florida, HM 1823, lacking the *Mi* gene, was used in all experiments. Seedlings were grown in black plastic trays with 32 cells (each cell 100  cm^3^ soil capacity). Sterilized potting mix and steam sterilized sand (1:1) was used to grow the seedlings. Four-week-old seedlings were used for all the experiments.

Southern root-knot nematode, *M. incognita*, was used in all the experiments. A pure *M. incognita* culture was developed by single egg mass inoculation approach and cultures were maintained on the RKN susceptible tomato cultivar HM 1823 in the greenhouse. RKN second-stage juveniles (J2s) (for growth room experiments) or freshly extracted eggs (for greenhouse experiments) were used as inoculums, eggs were extracted from 2 month-old tomato roots using 10% solution of commercial bleach (4% NaOCl) followed by sugar flotation using 40% sugar solution (Hussey and Barker, 1973). For J2 collection, egg suspensions were poured into modified Baermann bowls with ten layers of tissue wipes (40 cm × 40 cm × 0.03 cm, Kimwipes^®^, Kimberly-Clark Corporation, Irving, TX, USA) to ensure clean J2s. The bowls were covered with aluminum foil to simulate dark conditions in the soil, and some holes were made in the foil for air exchange. The bowls were incubated at 26°C, and J2s were obtained 5 to 7 days after incubation and used for inoculation. RKN, either J2s or eggs, were inoculated immediately after transplanting by pipetting equal suspension volumes (1 ml) into three 2.5 cm deep holes, 10 cm in diameter around the seedling.

*Growth room and greenhouse setting:* Greenhouse and growth room benches were sterilized with bleach. In the growth room, 25°C room temperature and 40% relative humidity was maintained. Light-dark cycle was 16 and 8 hr, respectively. Natural light with 26°C ± 2°C temperature and 60% relative humidity was maintained in the greenhouse.

*Growth room experiment to determine optimal concentration and timing:* The experiment was conducted to test the efficacy of three different concentrations of NAD (0.01  mM; 0.1  mM; 1  mM) against *M. incognita* on tomato cv. HM 1823. These three concentrations were evaluated for different application timings: pre (1 day before J2 inoculation, all three concentrations), pre+post (1 day before and 1 day after J2 inoculation, all three concentrations), and post (1 day after J2 inoculation, only for 1  mM). Water was used as a negative control. Four-week-old seedlings, as described above, were drenched with 10 ml NAD/pot and ~350 J2/pot of *M. incognita* were inoculated 1 day after NAD application. NAD post drenching was done 1 day after J2 inoculation. The experiment was conducted in a completely randomized design in the growth room, as described above, and was repeated.

Each treatment had 10 replications. Five plants from each treatment were sub-sampled to observe root penetration at 2 days after inoculation, and the remaining 5 plants were sub-sampled at 30 days after inoculation to make root gall ratings, count egg masses, and determine shoot and root dry weight. J2 penetration in the root system was examined by staining them inside the root using acid fuchsin (Byrd et al., 1983; Thies et al., 2002). Egg masses were stained using red food dye protocol developed by Thies et al. (2002). Root gall ratings were done on a scale of 0 to 10 (0 = no galls, and 10 = 100% of roots galled) (Zeck, 1971). Roots and shoots were oven dried at 70°C for 5 days.

Using the same NAD concentrations (0.01  mM, 0.1  mM, 1  mM) a mortality assay was conducted to test any potential direct effect of NAD on J2s. Six-well plates were used with four replications for each treatment. Each well had 2 ml of three different NAD concentrations and water as a negative control. Approximately 800 J2 of *M. incognita* were incubated in each treatment solution under dark condition at room temperature for two days. Live and dead nematodes were counted under a dissection microscope and the percentage of dead nematodes was calculated. Straight or “J” shaped, non-motile juveniles were considered as dead. The experiment was repeated.

*Greenhouse experiment to evaluate the long-term effect of NAD on root-knot nematode and tomato growth:* Out of the three different concentrations tested in the first experiment, 1 mM concentration of NAD was selected and tested with three different application approaches pre (1 day before inoculation), pre + post (1 day before and 1 day after inoculation) and post (1 day after inoculation). Water was used as negative control. Ten liter capacity pots were filled with steam sterilized Gulf Coast Research and Education Center (GCREC) soil and 4 week-old seedlings were transplanted for the experiment. The soil was sterilized for 12 hr at 70°C using SST-15 1/8 cubic yard 120v Soil Sterilizer (Pro-Grow Supply Corp, Brooksville, WI, USA. Approximately 18,500 freshly extracted *M. incognita* eggs were inoculated in each pot as described earlier. Fertilizer (20:20:20) at the rate of 2.7 g per liter was applied weekly before fruiting stage and calcium sulphate @ 0.5  g/liter was also applied during fruiting stage. Both fertilizer solutions were applied @ 200 ml/pot. Plant height was recorded bi-weekly; root gall rating, egg extraction, root and shoot drying was done as described earlier and recorded at 2 time points (60 and 105 days after transplanting; DAT). Yield was recorded biweekly at 3 time points (78, 92 and 105 DAT). The experiment was conducted in a completely randomized design and repeated.

*Field experiment:* The experiment was conducted at GCREC, Wimauma, Florida. The spring trial was conducted between February 26, 2020 and June 10, 2020. A similar fall trial was carried out between September 3, 2020 and December 9, 2020. The field was known to have a high natural population of *M. javanica*. The soil at GCREC was classified as Myakka fine sand (Sandy, Siliceous Hyperthermic Oxyaquic Alorthod) having 0.8% organic matter with pH of 7.6. The experiment was conducted to test the efficacy of NAD transplant applications to manage root-knot nematode in a field naturally infested with RKN, both in fumigated and non-fumigated beds. A RKN susceptible and popular tomato cultivar in Florida, cv. HM1823, was used in the experiment.

Tomato seedlings were treated in transplant trays with three different concentrations of NAD (1  mM, 5mM and no NAD for spring and 5mM, 10mM and no NAD for fall) and transplanted in beds that were treated as follows: (i) chloropicrin (100%) (Pic100^®^, TriEst Ag. Group, Tifton, GA) @ 225 kg/ha, (ii) 1,3-dichloropropene (40%) + chloropicrin (60%) (Pic-Chlor 60^®^, TriEst Ag. Group, Tifton, GA) @ 336 kg/ha, and (iii) no fumigant. The experiment was conducted in raised beds covered with black, totally impermeable film (TIF, Total Blocked, Berry Plastics Corporation, Evansville, IN) during spring and white TIF during fall. The experimental design was two-factorial split plot randomized block design, (i) Fumigant as the main plot, and (ii) NAD treatments as randomly assigned sub-plots within each fumigated and non-fumigated plots.

Tomato seedlings were grown at GCREC as described by Regmi and Desaeger (2020). Six-week-old tomato seedlings were treated with two different concentrations of NAD in each trial, 1 mM and 5 mM during spring and 5 mM and 10 mM during fall, two days prior to transplanting. Each seedling was drenched with approximately 10 ml solution. NAD-treated seedlings were then hand transplanted in 6 m long × 0.75 m wide size sub-plot at 46 cm apart totaling 13 plants per experimental plot. Fumigation, regular pest and disease management, irrigation and fertilizer application were done following GCREC standard recommendations.

Plant vigor was recorded at 28, 43, 55, and 69 DAT during spring and at 27, 41, 57, and 71 DAT during fall using a hand held crop sensor (GreenSeeker™, Trimble, Sunnyvale, CA). Root gall rating and soil RKN J2 population were determined at 69 and 105 DAT during spring and at 53 and 97 DAT during fall. Five plants were randomly sub-sampled for mid-season and end-of-season gall ratings in both seasons. Five soil cores were taken from each experimental plot directly from the root zone of the previous uprooted plants. Galls were rated on a 0–10 scale developed by Zeck (1971) where 0 means no galls and 10 means the whole root system is covered with galls and with no fibrous roots present. For soil nematode extraction, each soil sample was thoroughly mixed and 200 cc soil was sub-sampled and nematodes were extracted with an IKEA salad spinner 10 cm in diameter, lined with IKEA tissue paper and incubated for 48 hr.

Tomato fruits were hand-picked, graded and weighed at biweekly intervals at three times from eight plants. Total yield was determined by combining yields for all harvest times.

### Data analysis

Data obtained were examined by analysis of variance (ANOVA) for a factorial design using PROC GLIMMIX procedure of SAS version 9.4 (SAS Institute Inc., NC). Means of data were separated by using LSMEANS statement in SAS at *P* ≤ 0.05. Due to high variation, juveniles and egg count data were analyzed using negative binomial approach in SAS. The data from 2 runs for each experiment (growth room and greenhouse) were combined since there was no significant interaction between run and treatments.

## Results

*Growth room experiments:* All application timings of 1mM NAD (pre, pre + post or only post inoculation) significantly suppressed J2 root penetration compared to the non-treated control at 2 DAI (Table 1). Among three different NAD concentrations tested, 0.1 mM NAD when applied pre or post and 0.01 mM NAD when applied pre + post also suppressed J2 penetration significantly. All NAD concentrations for all application timings suppressed egg masses at 30 DAI compared to the control (Table 1). However, only post application of 1  mM NAD significantly suppressed root gall ratings compared to the control at 30 DAI. Shoot weight was significantly increased for 1  mM NAD as compared to the control treatment at 30 DAI for all application timings (Table 1). Root weight was significantly higher in the plants treated with pre applied 1  mM NAD (Table 1).

**Table 1.** Effect of Nicotinamide adenine dinucleotide (NAD) on root-knot nematode, *Meloidogne incognita* infection and plant growth on tomato (*Solanum lycopersicon* L.) cultivar HM 1823 in growth room experiment, Sep-Dec, 2018.

NAD treatments	J2 penetration (2 DAI)	Gall rating (0-10) (30 DAI)	Egg mass/g root (30 DAI)	Shoot weight (g) (30 DAI)	Root Weight (g) (30 DAI)
Control	85a	3.3ab	159a	3.93c	0.74bcd
1 mM Pre	41d	3.1abc	88c	4.74a	0.86a
0.1 mM Pre	61bc	2.8bc	77c	4.38abc	0.80abcd
0.01 mM Pre	74ab	3.6a	98c	4.11bc	0.70d
1mM Pre + Post	47cd	2.9bc	129b	4.85a	0.81abc
0.1 mM Pre + Post	73ab	2.7bc	100bc	4.15bc	0.73cd
0.01 mM Pre + Post	45cd	3.4ab	104bc	4.35abc	0.81abc
1 mM Post	48cd	2.6c	96c	4.56ab	0.83ab
*P-*value	<.0001	0.026	0.0003	0.0071	0.03

Note: DAI = days after inoculation; Pre = NAD treatment one day before transplanting; Post = NAD treatment one day after transplanting. Factor levels sharing the same letter do not differ significantly (*P*-value > 0.05), according to Tukey’s HSD.

In the mortality assay none of the concentrations (0.01  mM, 0.1  mM and 1  mM NAD) showed any difference in J2 mortality as compared to the control (all around 2% mortality, data not shown).

**Figure 1:**
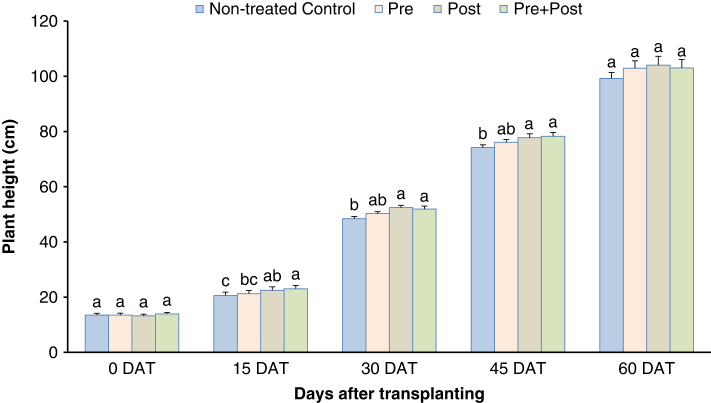
Effect of 1mM Nicotinamide adenine dinucleotide (NAD) applied as a pre-, post- or pre+post- plant drench application on plant height of tomato (*Solanum lycopersicon* L.) cv. HM1823 in the greenhouse; pre = NAD treatment 1 day before transplanting; post = NAD treatment 1 day after transplanting; pre+post = NAD treatment 1 day before and 1 day after transplanting; DAT = days after transplanting. Factor levels sharing the same letter within each time point do not differ significantly (*P*-value > 0.05), according to Tukey’s HSD.

*Greenhouse experiments:* Based on the previous assay, 1mM NAD was selected as the concentration to use in the greenhouse trial to evaluate the season-long effect of NAD on RKN control, tomato growth and yield (Fig. 1, Table 2). Pre + post application of NAD consistently gave greater plant height from 15 days after transplanting (DAT) until 60 days DAT, the difference being statistically significant at 15, 30 and 45 DAT (Fig. 1). Gall ratings were suppressed by 15 to 27% for all NAD treatments at 60 DAT compared to the control but at the end of the trial at 105 DAT no more differences were noted. Only pre + post NAD application slightly suppressed gall rating at the end of the trial (Table 2). Nematode reproduction in terms of eggs/g root was significantly lower on plant roots with pre + post NAD treatment at 60 DAT but no more difference was noted at 105 DAT. No treatment effect was noted on shoot and root dry weight at 60 and 105 DAT, or on tomato yield (Table 2).

**Table 2.** Effect of 1mM Nicotinamide adenine dinucleotide (NAD) on root-knot nematode, *Meloidogne incognita* severity and plant biomass of tomato (*Solanum lycopersicon* L.) cultivar HM 1823 in the greenhouse, Gulf Coast Research and Education Center (GCREC), Feb–May, 2019.

	Gall rating (0–10)		Eggs/g root		Dry root weight		Dry shoot weight		
Treatments	60 DAT	105 DAT	60 DAT	105 DAT	60 DAT	105 DAT	60 DAT	105 DAT	Total fruit yield (kg)
Control	4.0a	8.3a	54688a	291536a	3.76a	4.41a	26.12a	54.21a	1.29a
Pre + Post	2.9b	7.2b	27393b	269600a	4.00a	4.88a	28.99a	53.83a	1.28a
Post	3.3b	8.1a	41353a	276340a	3.70a	5.21a	27.89a	60.48a	1.26a
Pre	3.4b	8.0a	45705a	278300a	4.33a	4.67a	28.40a	57.96a	1.25a
*P-*value	0.0021	0.0001	0.007	0.89	0.214	0.833	0.230	0.507	0.986

Note: DAT = days after transplanting; Pre = NAD treatment one day before transplanting; Post = NAD treatment one day after transplanting. Factor levels sharing the same letter do not differ significantly (*P*-value > 0.05), according to Tukey’s HSD.

**Figure 2:**
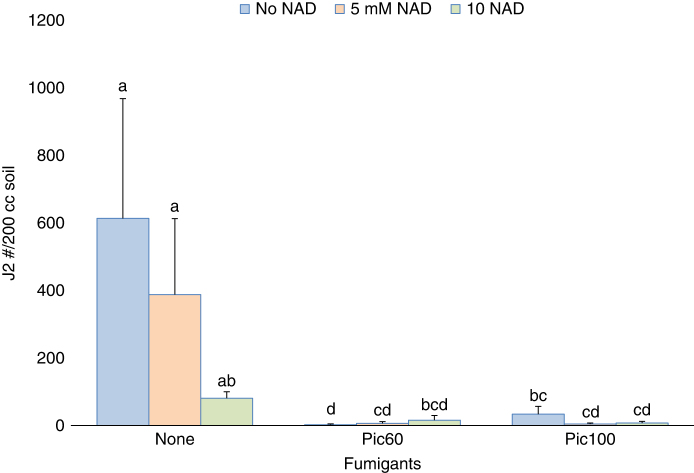
Effect of Nicotinamide adenine dinucleotide (NAD) on soil root-knot nematode, *Meloidogne incognita* population in fumigated and non-fumigated beds, field trial, fall, 2020. Factor levels sharing the same letter do not differ significantly (*P*-value > 0.05), according to Tukey’s HSD.

*Field experiments:* During spring, neither 1 mM nor 5 mM reduced root gall ratings at the middle or end of the season, or significantly affected soil RKN populations at the end of the season (Table 3). In fall, 5 mM and 10 mM NAD also did not have any significant impact on reducing RKN population (Table 4), but in non-fumigated beds a trend towards lower root-knot nematode soil populations was observed in NAD treated plots (Fig. 2). Fumigants had a significant effect on RKN in both seasons. In spring, root gall ratings were significantly suppressed by both fumigants at 69 DAT, but only 1, 3-D + chloropicrin reduced root gall ratings and soil RKN counts at 105 DAT (Table 3). During fall, both fumigants reduced root galling and soil RKN counts both at mid-season and after final harvest (Table 4).

**Table 3.** Root gall ratings of tomato (*Solanum lycopersicon* L.), root-knot nematode, *Meloidogne incognita* population in soil, and tomato fruit yield as affected by fumigants and Nicotinamide adenine dinucleotide (NAD), field trial-spring 2020, Gulf Coast Research and Education Center (GCREC), FL.

		Root-knot severity (0–10 scale)		J2s/200 cc soil		Tomato Yield (kg/plot)		
Factors	Level	69 DAT	105 DAT	105 DAT	77 DAT	94 DAT	103 DAT	Total yield
	None	3.23a	5.77a	60a	16.96b	12.86a	5.13a	34.96b
Fumigant	Pic 100	1.65b	5.34a	82a	22.92b	13.78a	5.44a	42.15a
	Pic-Clor 60	0.08c	0.53b	7b	21.67a	12.44a	6.23a	40.35ab
	None	1.16a	4.16a	46a	19.76a	12.43a	7.06a	39.26a
NAD	1mM	1.89a	3.27a	49a	20.06a	13.40a	5.54a	39.20a
	5 mM	1.91a	4.22a	55a	21.73a	13.26a	4.20a	39.01a
	Fumigant	<.0001	<.0001	0.0005	0.005	0.796	0.763	0.106
*P*-value	NAD	0.290	0.535	0.423	0.459	0.875	0.201	0.996
	Fumigant*NAD	0.824	0.678	0.366	0.105	0.500	0.738	0.322

Note: DAT = days after transplanting. Factor levels sharing the same letter within each factor do not differ significantly (*P*-value > 0.05), according to Tukey’s HSD.

**Table 4.** Root gall ratings of tomato (*Solanum lycopersicon* L.), root-knot nematode, *Meloidogne incognita* population in soil, and tomato fruit yield as affected by fumigants and Nicotinamide adenine dinucleotide (NAD), field trial-fall 2020, Gulf Coast Research and Education Center (GCREC), FL.

		Root-knot severity (0–10 scale)		J2s/200 cc soil		Tomato Yield (kg/plot)		
Fumigant	NAD	53 DAT	97 DAT	97 DAT	68 DAT	82 DAT	96 DAT	Total yield
	None	3.28a	5.24a	361a	1.56c	5.99b	9.90a	17.46b
Fumigant	Pic 100	0.26b	0.46b	15b	5.51b	10.53a	11.06a	27.11a
	Pic-Clor 60	0.06b	0.15b	8b	8.79a	8.51ab	12.45a	29.76a
	None	1.04a	2.04a	217a	5.06a	8.53a	12.13a	25.74a
NAD	5 mM	1.51a	2.20a	133a	5.14a	7.47a	10.65a	23.27a
	10 mM	1.06a	1.62a	35a	5.66a	9.03a	10.63a	25.32a
	Fumigants	<.0001	<.0001	<.0001	<.0001	0.024	0.160	<.0001
*P*-value	NAD	0.474	0.409	0.591	0.763	0.569	0.412	0.393
	Fumigants*NAD	0.275	0.213	0.060	0.824	0.798	0.254	0.758

Note: DAT = days after transplanting. Factor levels sharing the same letter within each factor do not differ significantly (*P*-value > 0.05), according to Tukey’s HSD.

**Figure 3:**
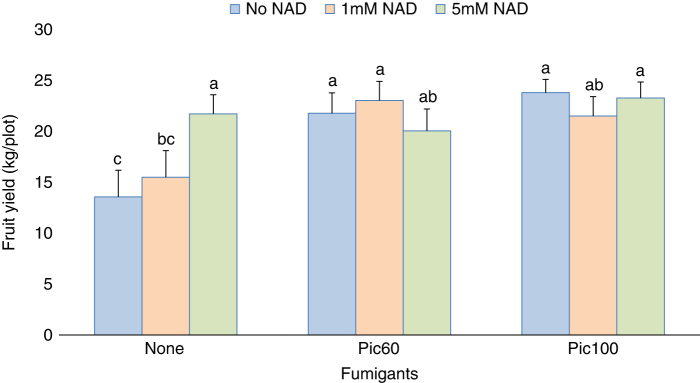
Effect of Nicotinamide adenine dinucleotide (NAD) on early tomato (*Solanum lycopersicon* L.) fruit production at 77 DAT in fumigated and non-fumigated beds; field trial spring 2020; DAT = days after transplanting. Factor levels sharing the same letter do not differ significantly (*P*-value > 0.05), according to Tukey’s HSD.

Plant stand counts were not affected by any of the treatments, as was scattered plant mortality caused by southern blight (*Athelia rolfsii* (Curzi) C.C. Tu & Kimbr) (data not shown). Tomato plant vigor throughout the season was not improved by any of the NAD treatments in both seasons. Early plant vigor was better in fumigated beds in both seasons but no treatment effect on plant vigor was noted after 40 days (data not shown). Especially early yield was significantly increased in fumigated- as compared to non-fumigated beds in both seasons (Tables 3 and 4). NAD treatments did not show a significant impact on total fruit yield in both seasons. However, during spring, while NAD did not affect early tomato fruit yield in fumigated beds, the 5 mM NAD did increase early fruit yield significantly (by 24%) in non-fumigated beds (Fig. 3).

## Discussion

NAD can protect crop plants from different biotic and abiotic stresses by inducing plant innate immunity. In general, pyridine nucleotides, such as NAD, are involved in calcium (Ca^2+^) signaling and DNA repair in plants via poly-ADP-ribosylation and protein deacetylation (Pétriacq et al., 2013). Recently, some studies have shown that exogenous application of NAD or its precursors can induce plant defense mechanisms against certain fungal and bacterial pathogens in the model plant *Arabidopsis thaliana* (L.) Heynh (Zhang and Mou, 2009; Pétriacq et al., 2012; Pétriacq et al., 2016; Miwa et al., 2017; Alferez et al., 2018).

In previous studies, we demonstrated that exogenous application of 5mM NAD can induce plant defenses in tomato against the root-knot nematode species, *M. hapla* and suppress nematode root invasion, root galling and egg production, and at the same time stimulate plant biomass (Abdelsamad et al., 2019). The studies reported here demonstrate that, in both growth room and greenhouse experiments, 1mM NAD provided short term protection against *M. incognita* and enhanced plant growth of tomato. These findings are supported by our previous study where an application of 5 mM NAD resulted in significantly less J2 penetration at 2 DAI, fewer root galls at 30 DAI and better plant growth compared to the non-treated control (Abdelsamad et al., 2019). None of the three NAD concentrations showed any direct nematicidal effect, confirming that the NAD effect is indirect by stimulating plant defenses (Abdelsamad et al., 2019). Nicotinamide mononucleotide (NMN), a precursor of NAD also did not show antifungal properties when tested against a fungal disease of barley (*Hordeum vulgare* L.) *Gibberella zeae* (Schwein.) Petch. Disease alleviation in *Arabidopsis* leaves and flowers and barley spikes was solely based on induced salicylic acid (SA)-dependent and SA-independent signaling pathways (Miwa et al., 2017).

However, NAD benefits were mostly short-term. In the greenhouse trials, 1mM NAD applied just before or after nematode inoculation suppressed root galls at 60 days, but not after 105 days. Interestingly, applying NAD twice provided stronger and longer protection against RKN. Two applications of 1 mM NAD (just before and after inoculation) suppressed root galls up to 105 days, and unlike the single NAD applications, it also suppressed root-knot egg production after 60 days. Multiple applications (4–5 times) of Actigard^TM^ (acibenzolar-S-methyl (ASM), the first commercially available chemical elicitor, were also necessary to get desired control over *Phytophthora* root and crown rot in pepper (*Capsicum annum* L.) plants equivalent to the chemical approach ((Matheron and Porchas, 2002). Similarly, our colleagues at the Citrus Research and Education Center (CREC, Lake Alfred, FL) reported that 1 and 5 mM NAD induced resistance equivalent to 2ml/liter Actigard^TM^ solution against citrus canker disease in a greenhouse study (Alferez et al., 2018).

Despite the observed suppression in (early) nematode damage, none of the NAD applications in our greenhouse trial had a significant effect on tomato growth and fruit yield. Probably the level of root-knot damage, especially at the end of the trial, did not show enough difference among treatments to result in differences in and affected tomato growth.

In the two field trials, we wanted to evaluate if treating tomato seedlings in the transplant house prior to planting in a field naturally infested with *M. javanica* could provide any benefits. Because fumigation prior to planting is standard practice among tomato growers in Florida, we tested NAD-treated plants in fumigated beds as well as in non-fumigated conditions. Both fumigant treatments in our trials, chloropicrin only, and chloropicrin combined with 1,3-D, significantly reduced nematode infection and soil populations as expected. Combinations of 1,3-D and chloropicrin are the standard practice in Florida tomato fields, as they have been shown to give good control of both RKN and soilborne pathogens (Santos et al., 2006; Minuto et al., 2006; Desaeger et al., 2017). NAD-treated plants did not offer much benefit in fumigated beds, both in terms of root-knot nematode control, or tomato crop vigor or fruit yield. The fumigants, especially the 1,3-D + chloropicrin treatment, gave very effective nematode control and consequent yield benefit, so there was limited scope for further benefits from NAD treatments. However, NAD-treated plants did show some positive effects in non-fumigated beds. In spring, early tomato yield in non-fumigated beds was increased by 24% for 5 mM NAD-treated plants as compared to non-treated plants. In fall, RKN soil counts in non-fumigated beds were suppressed (*P =* 0.10) for seedlings that were treated with 10 mM NAD. The benefit of NAD therefore seems to be more apparent in non-fumigated conditions. Whether this is simply because the potential for providing benefit is less in fumigated beds, or because the effect of NAD on inducing a plant defense response is negatively affected by fumigation, was not determined. These field results, however, do show that treating tomato seedlings with NAD in transplant houses and prior to planting, may still provide a benefit in the field, and especially in non-fumigated fields. As such, NAD, and possibly other plant resistance activators, can have potential to become a component of more integrated non-fumigant nematode management programs. Fumigants are still the primary nematode management practice in Florida tomatoes, as well as in most other high-value crops in the state. However, an increasing number of growers are starting to move away from fumigants since new more selective and less toxic nematicides like fluensulfone and fluopyram have become available. More nematicides will become available in the next years, and it will be interesting to determine how NAD and other potential systemic transplant treatments could be combined with these new nematicides. It is very likely that an increasing number of growers in Florida will continue to adopt non-fumigant soil management programs. How fast this will happen will be impacted by regulatory changes, in addition to viable alternatives.

Success of plant activators to induce systemic acquired resistance in crop plants are mostly in controlled conditions and their efficacy in the field depends on several factors like dose, plant species and cultivars, growth stage of the plant, pathogen pressure and climatic condition (Gozzo and Faoro, 2013). Our understanding of the impact of these factors on the efficacy of resistance-inducing agents is still rudimentary (Walters and Fountaine, 2009). As stated earlier, multiple applications may be needed for effective nematode management in the field, in which cost will be a deciding factor.

More research is needed, but based on this and other studies, NAD is a potential next generation plant immunity elicitor which could be successfully incorporated in integrated pest management programs as a plant defense activator against a range of pest and pathogens important in agriculture, including root-knot nematodes and possibly other tomato pathogens. It would be especially valuable to look into combinations of plant defense activators like NAD with new chemical and biological nematicides, and in case of tomatoes, also *Mi*-resistant cultivars.
